# Cutaneous Intravascular NK/T-cell lymphoma mimic panniculitis clinically, case report and literature brief review

**DOI:** 10.1186/s13000-015-0330-0

**Published:** 2015-07-16

**Authors:** Ahmed Alhumidi

**Affiliations:** Department of Pathology, College of medicine, and king Khalid university hospital, King saud university, P.O. BOX 2925, Riyadh, 11461 Saudi Arabia

## Abstract

Intravascular large cell lymphoma is a rare subtype of extranodal large cell lymphoma characterized by the presence of neoplastic cells within the lumina of small vessels. Most cases of intravascular large cell lymphoma have a B-cell phenotype. To date, 12 cases of intravascular natural killer (NK/)/T–cell lymphoma (IVNKL) have been reported. Our case is A 47-year-old female presented with erythematous patches and plaques on the lower extremities mimicking panniculitis clinically. A skin biopsy revealed intravascular lymphoma (IVL) with a NK/T cell phenotype (positive for CD3, and granzyme B and negative for CD20, CD4, CD8, CD5). The lymphoma cells were also positive for Epstein-Barr virus by Epstein-Barr virus-encoded RNA in situ hybridization test. Because this type of lymphoma is extremely rare, our case is documented and compared with the previously reported cases.

## Background

Intravascular lymphoma (IVL) is a rare form of non-Hodgkin lymphoma characterized by massive proliferation of large, neoplastic cells in small- and medium-sized blood vessels with a predilection for the skin and the central nervous system. This disorder was first described as angioendotheliomatosis proliferans systemisata by Pfleger and Tappeiner [1] in 1959. Most cases of IVL are of B-cell immunophenotype; rare cases of NK/T-cell IVL have been reported. To date, 12 cases of intravascular natural killer (NK)/T–cell lymphoma (IVNTKL) have been reported, our case is the 13^th^. Because this variant is extremely rare, our case is documented and compared with the previously reported cases

## Case presentation

**Fig. 1:**
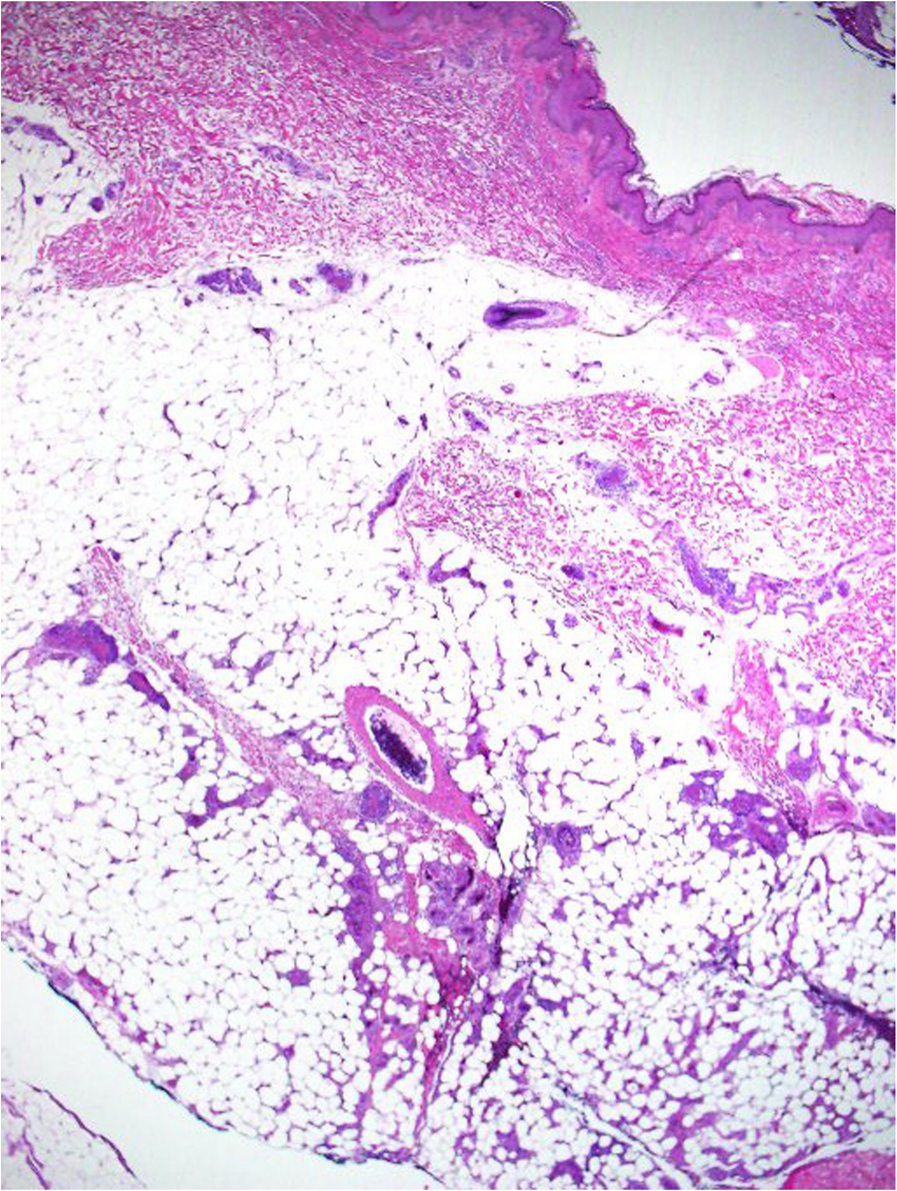
Scanning view of the cutaneous lesion revealing subcutaneous abnormal obstructed vessels (H/E, ×20)

**Fig. 2:**
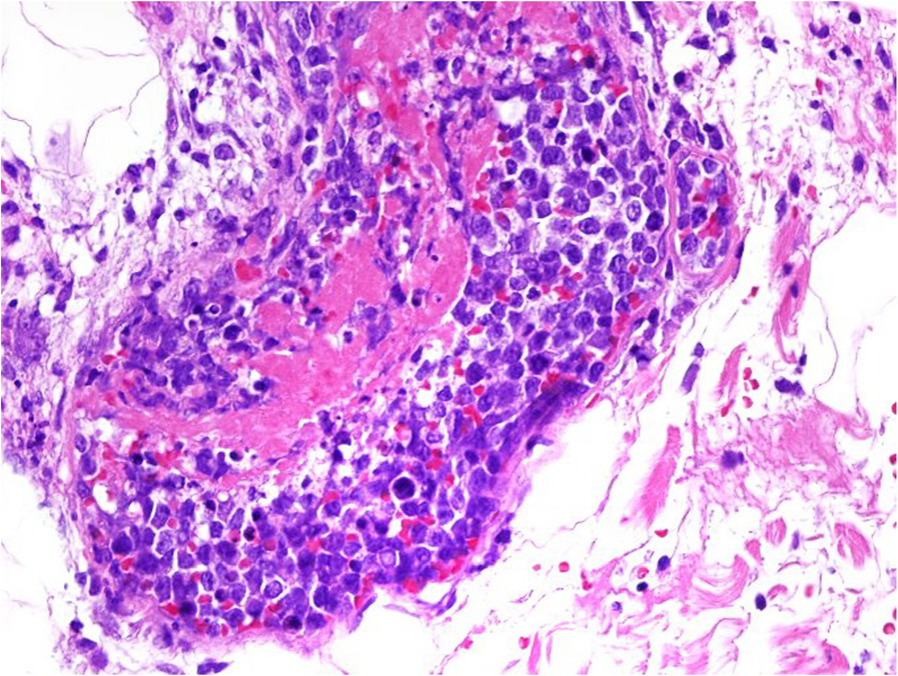
Higher magnification showing distended vessels containing atypical lymphoid cells with focal fibrin thrombi (H/E, ×400)

**Fig. 3:**
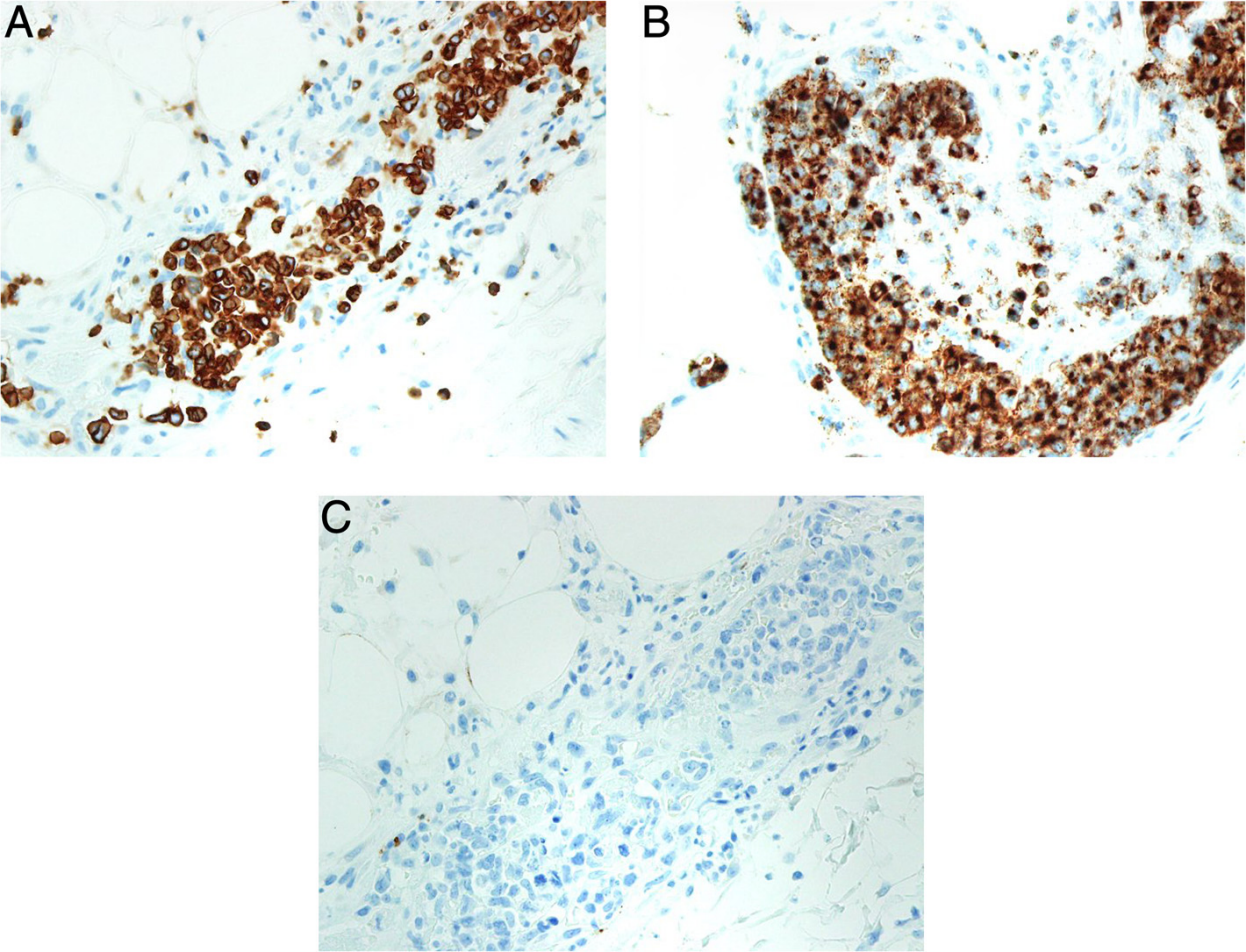
The intravascular lymphoma cells were CD3+ (**a**), granzyme B **b**. and negative for CD20 (**c**) (immunoperoxidase hematoxylin counterstain; ×200)

**Fig. 4:**
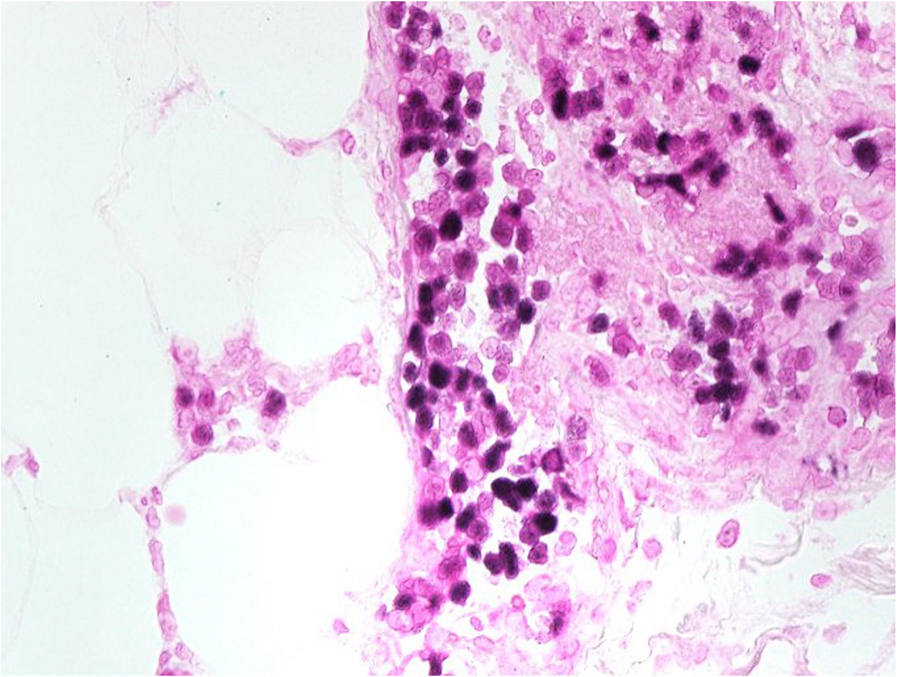
The lymphoma cells were positive for EBV by EBV-encoded RNA in situ hybridization study (in situ hybridization ×200)

A 48-year-old woman visited a dermatology clinic complaining erythematous patches and nodules on her lower extremities noted for 3 months. The lesions were not painful or pruritic. The results of all laboratory studies, including CBC count, liver function tests were unremarkable. Her medical and family histories were unremarkable. Under the impression of panniculitis, a skin biopsy was taken from a lesion on her leg. The histological study revealed many distended vessels filled with atypical large lymphoid cells in the subcutaneous tissue (Fig. 1). The tumor cells were all confined to the vessels and had large, irregular hyperchromatic nuclei with an ample eosinophilic cytoplasm (Fig. 2). Many mitotic figures and necrosis were observed. Immunohistochemical studies showed that tumor cells were CD45+ CD3+ (Fig. 3-a), Granzyme B+ (Fig. 3-b), CD56−, CD4−, CD5−, CD8−, CD20− (Fig. 3c), CD30−. In situ hybridization for EBER was positive (Fig. 4). The immunophenotype suggested that the intravasacular cells were NK/T-cell–like lymphoid cells. Bone marrow biopsy and aspirate revealed no evidence of tumor involvement. The patient did not have nasal lymphoma. The absence of peripheral leukemia and the lack of bone marrow involvement excluded the possibility of an aggressive NK-cell leukemia. The immunohistochemical tests and EBER in situ hybridization as well as the clinical finding suggested IVNKTL. After this diagnosis, Computed tomography scan and magnetic resonance imaging (MRI) of the brain, chest and abdomen were done and were unremarkable. Following combination chemotherapy the patient was alive with no evidence of disease at 18 months.

## Conclusions

IVL is a very rare neoplasm characterized by proliferation of lymphoma cells almost exclusively within the blood vessels. Originally described in 1959 as “angioendotheliomatosis proliferans systemisata” and considered an endothelial neoplasm, IVL has subsequently been shown to be a lymphoid malignancy based on immunohistochemical techniques [1–3]. The majority of reported cases were large B-cell lymphomas, only a few cases were reported to be of the T and NK-cell lineage. Since 2003, 12 cases of IVNKL have been reported worldwide. The current case is the 13th.

Recognized variants of NK cell lymphomas include a clinicopathologic spectrum of predominantly extranodal disease, including nasal and extranasal types, and aggressive NK cell leukemia/lymphoma [3, 4]. IVNKL should be distinguished from the extranodal NK/T-cell lymphoma, nasal type (ENKTCL). Although ENKTCL can also occur in the skin but presents with multiple nodules with ulceration and the tumor cells are distributed in tissues and show vascular invasion. Patients with IVNKTL have no nasal abnormalities and tumor cells are confined to the endovascular system. IVNKTL should also be distinguished from aggressive NK-cell leukemia. A rash is typically prominent in IVNKTL without obvious abnormalities in the peripheral blood, although bone marrow abnormalities might be present in some patients. In aggressive NK-cell leukemia, tumor cells are diffusely scattered in the extravascular tissue rather than deposited in blood vessels.

Why are all tumor cells distributed intravascularly in IVNKTL? The lack of adhesion molecule (CD29 and CD54) expression by tumor cells in intravascular large B-cell lymphoma is thought to explain why these tumor cells do not infiltrate extravascular sites [4]. Whether similar mechanisms exist in IVNKTL is not yet known. The high prevalence of EBER positivity in IVNKTL suggests that EBV infection is somehow involved in the pathogenesis of this rare lymphoma. Because these are rare cases, the origin of intravascular NK/T-cell lymphoma has not been studied in depth. Only two of the 12 cases had T-cell receptor gene rearrangement analysis performed, which confirmed NK/T-cell origin [5].

The age range of the reported IVNKL cases was between 4–71 years. The clinical course lasted from 2 months to 3 years. Multisystem involvement (brain, bone marrow, spleen, and kidney) occurred in eight of 13 patients (61.5 %). The other five patients including our case developed only a skin rash. Among the 13 cases, four (30.7 %) were reported in China, two (15.3 %) in Taiwan, two (15.3 %) in the United States, one (7.6 %) in Austria, one 7.6 %) in Japan, one in Korea, one (7.6 %) in Italy and one in Saudi Arabia (7.6 %). Nine (69.2 %) of 13 of the reported cases were encountered in Asian countries. Nine out of 13 patients were female (69.2 %) and four were male (30.8 %) [4–15].

The prognosis for IVL in general might vary depending on the extent of disease. Patients with limited disease such as skin lesions, as noted in our case, or single organ involvement might respond well to chemotherapy. Patients with disseminated disease, particularly those with CNS involvement, have done poorly [4–15].

In conclusion, Although the majority of IVL cases are B cell in lineage, rare cases of T or NK-cell lymphoma can occur as IVL. Because this variant is extremely rare, our case is documented and pathologists should be aware that NK-cell lymphoma can also occur intravascularly as cutaneous lymphoma with or without affecting other internal organs. The diagnosis can be established by combined histopathologic, immunohistochemical, and molecular studies. The role of EBV in the oncogenesis of IVNKL requires further investigation. More cases should be documented for further understanding of this peculiar rare type of lymphoma.

## Consent

Consent was obtained from the patient for publication.
